# Ectopia lentis surgery in Marfan’s syndrome: from “couching” to the use of intracapsular tension rings

**DOI:** 10.22336/rjo.2022.2

**Published:** 2022

**Authors:** Anca Cristina Dogăroiu, Cătălin Dogăroiu, Călin Petru Tătaru

**Affiliations:** * Alcor Clinic, Bucharest, Romania; ** “Carol Davila” University of Medicine and Pharmacy, Bucharest, Romania; *** Clinical Hospital of Ophthalmologic Emergencies, Bucharest, Romania

**Keywords:** ectopia lentis, Marfan’s syndrome, surgical techniques

## Abstract

In the course of time, correction of the ectopia lentis in Marfan’s syndrome has been approached through quite many surgical techniques. They addressed the iris, or the lens and aimed at increasing the aphakic zone, the opacification or removal of the lens.

Although associated to many complications, the first techniques proved the courage and imagination of pioneer surgeons and are worth mentioning. They were steps towards the invention of current techniques and devices that make surgery of ectopia lentis safe, easy, and having a favorable prognosis.

## Introduction

Malpositioning of the lens was first described by Berryat in 1749, while the term ectopia lentis was introduced later, in 1856, by the Austrian ophthalmologist Stellwag [**1**].

With a long history and diverse etiology (trauma, pseudoexfoliative syndrome, systemic diseases or as a single entity), the association of ectopia lentis with Marfan’s syndrome was made only in 1923 by Ormond and Williams [**1**-**4**]. They reported that the patients suffering from arachnodactyly, one of the early terms defining the Marfan’s syndrome, also had a large range of eye problems, among which iridodonesis and ectopia lentis [**2**,**3**].

From the very beginning, the high frequency of this association has raised a controversy regarding the opportunity of the surgical approach, its timing, and the best suited technique. If Knapp and Chandler sustained that opportunity and timing of surgery should be done when vision became unsatisfactory - as described by Chandler under 20/ 70 [**5**,**6**], as for the elected surgical technique, lots of trials have been made. All these trials are history and, although technically old, they deserve to be mentioned as they lie at the basis of progress and modernization of surgical approaches to ectopia lentis.

## Materials and methods

A general presentation of surgical approaches in ectopia lentis, from its beginnings to current techniques, used in treating this pathology, is presented.

The first surgical techniques involved the iris, or the lens.

The techniques regarding the iris were mainly conservatory and aimed at enlarging the aphakic zone. On the other hand, lens techniques were versatile and aimed at enlarging the aphakic zone, or the complete removal of the lens.

In 1860, Crichett [**7**] worked out a technique aimed at enlarging the aphakic zone in patients suffering from ectopia lentis. The procedure, called iridodesis, followed the next steps: the cornea was punctured with a long needle, the iris was externalized at the level of this incision, leaving the pupillary edge of the iris within the anterior chamber. The operation was concluded by stitching the iris captured through the incision.

**Fig. 1:**
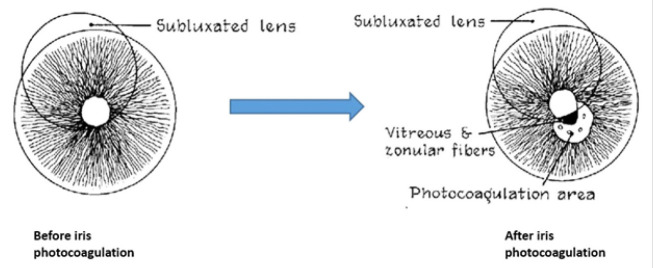
Aspect of iris before and after photocoagulation. Images reproduced after Straatsma’s “Lens Subluxation and Surgical Aphakia Treated with Photocoagulation of the Iris”

Straatsma [**8**] suggested other surgical approaches to the iris, i.e., the surgical removal of a part of the iris (iridectomy), or the photocoagulation of the iris. The latter entailed the modification of the pupil aperture to enlarge the aphakic zone (**Fig. 1**). This technique could be used only in particular cases in which the risk of severe complications was too high or when other eye problems that altered the size of the pupil were present.

The lens techniques were aimed at completely luxating the lens into the vitreous cavity, opacification of the lens by puncturing (discission) or the removal of the lens by means of a forceps or other devices.

“Couching”, the oldest method of cataract surgery, is an Indian technique that was no longer in use in cataract surgery, but still had indication in ectopia lentis [**9**]. The operation could be done both by anterior or posterior approach [**10**].

The anterior approach requires [**10**] the perforation of the cornea by means of a sharp instrument, which, after penetration, is directed towards the iris or, through the pupil, towards the periphery of the lens. Once in position, the surgeon changes the direction of the movement by raising the instrument and putting pressure on its tip, which was already placed on the lens capsule or within the lens. The lens was made to move back- and downwards, thus, freeing the pupil area.

The posterior approach goes as follows, i.e., a cutting instrument with a stopper (the stopper is made of cotton and prevents the instrument to go too deep) is introduced into the sclera, right behind the ciliary body. Then, a copper probe, also provided with a stopper, is introduced through the incision and, by circular movements, breaks the fibers of the suspensory ligament. When the fibers are broken, the tip of the instrument is pressed on the lens that was luxated into the vitreous cavity.

Another technique frequently employed in the beginnings of ectopia lentis surgery was discission. This approach was first described by Sattler in 1897 [**5**,**11**] and consisted in the perforation of the eye with a knife and then of the lens capsule with a needle. Thus, the lens with its capsule broken undergoes a process of opacification and shrinking that leads to the enlargement of the pupil aphakic zone. In order to hasten the opacification process of the lens, some surgeons improved Sattler’s initial technique. So, Knapp suggested [**5**] the perforation of the lens in the area opposed to the subluxation in two places at the level of the sclera by means of two knives, so, the instruments used to penetrate the lens in the same place were the two knives placed one upon the other, handled in such a way as to enlarge the intracapsular opening and generate mobilization of the lens material. The result was the opacification of the lens and the enlarging of the aphakic zone.

The techniques described by Chandler [**6**] and Kravitz [**12**] required two needles at the same time. One was used to stabilize the lens, and the second to perforate it. The authors believed that these techniques were gentler than Knapp’s approach, because, by using the supporting needle, fewer tractions on the ciliary body were registered. However, discission, irrespective of the chosen technique, was complicated and often required further surgery to induce adequate opacification of the lens. Therefore, some authors sustained the idea of completely removing the lens.

**Fig. 2:**
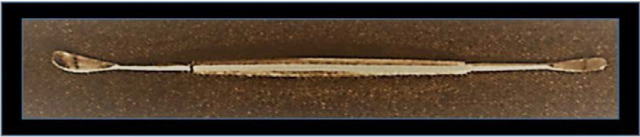
Spatula or Smith’s spoon. Images reproduced after Vale’s “Surgery of the Subluxated Lens”

Smith suggested the removal of the lens and its capsule [**13**]. This approach [**13**] requested the use of a special instrument, later called Smith’s spatula (**Fig. 2**): a circumferential incision was made in the zone opposed to the subluxation and two threads of corneo-scleral suture were made. The part of the iris at this level was removed (iridectomy) and the spatula was introduced under the lens. The latter was raised to meet the internal face of the cornea, and with the spatula maintained in this position, exterior pressure was put by means of a hook or of a loop placed in the subluxation zone. As a result of this pressure, the lens slipped on the smooth surface of the spatula, like on a runway, without putting any pressure on the vitreous body. The operation was concluded by tying up the two threads placed first. Employing this technique, the loss of vitreous matter was minimal or null.

In subluxated lenses, Kirby employed the technique of intracapsular extraction that did not imply exterior pressure [**14**]: corneal threads were placed first, followed by an upper limbic incision and then iridotomy; raising the central suture thread, the capsule forceps was introduced into the eye and fixed on the anterior capsule; gently pulling downwards the lens, zonular fibers were more clearly visualized and detached from the capsule with a special hook invented by Kirby; the lens was extracted and the operation was concluded by tying up the threads.

**Fig. 3:**
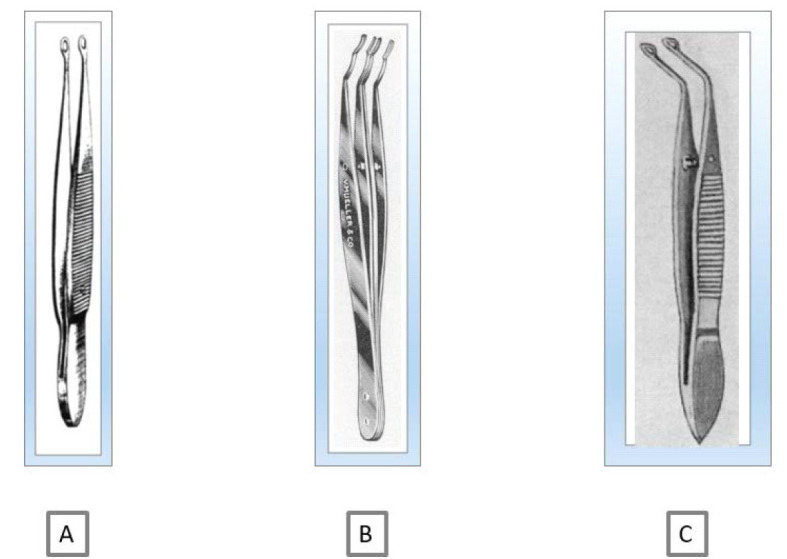
Forceps types used to grasp the lens **A.** Forceps devised and described by Walker E. Charles in “A Modified Capsule Forceps for Cataract Extraction”; **B.** Forceps devised and described by Cullom M. in “A New Capsule-Grasping Forceps”; **C.** Forceps devised and described by Berens Conrad in “A New Capsule Forceps”

Over the years, several lens extraction techniques by means of forceps have been described. These techniques varied according to the grasping of the lens (the whole lens, only the front lower capsule, only the front upper capsule) or through slight modifications of forcepses (**Fig. 3**) [**15**-**19**]. Among the users of this technique, the Romanian ophthalmologist Stanculeanu Gheorghe was mentioned in the literature of the time.

In 1911, Hullen invented and used a suction device for intracapsular removal of the lens [**20**,**21**]: cocaine anesthesia of the eye was followed by the placing of a blepharostat to keep the eye open, and then, the surgeon performed a limbic circumferential incision that looked like a funnel, in which a silk thread was placed; an iridectomy was then performed; the blepharostat was removed and the eye was kept open by the assistant without any pressure on the eyeball; while the patient kept looking in primary position, the cup of the suction device was placed on the front capsule in the center of the lens; by activating the handle of the device, vacuum, which firmly caught the lens, was created; antero-posterior rotations detached the lens from the suspending ligament and then, the lens was gently raised and removed from the eye; the operation was concluded when the silk thread was tied up.

**Fig. 4:**
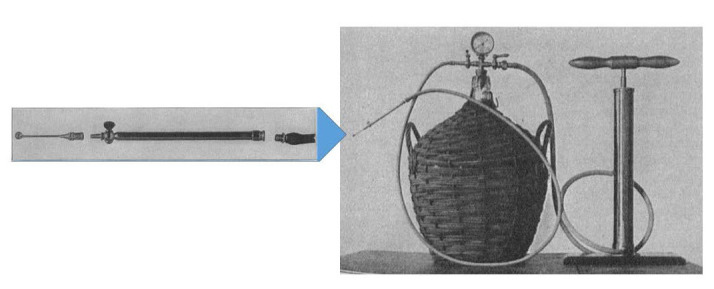
Lens suction device. Image reproduced after Hulen Vard’s “Vacuum Fixation of the Lens and Flap Suture in the Extraction of a Cataract in its Capsule”

**Fig. 5:**
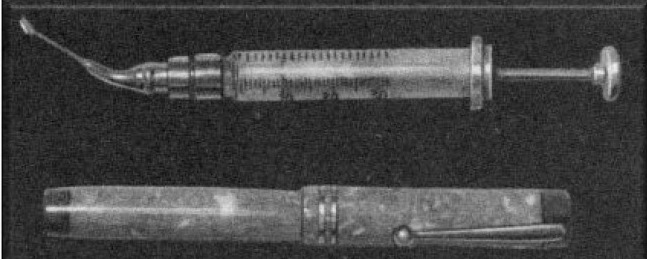
Erysiphake. Image reproduced after Dimitry J.’s “Evolution of a Sucking Disk for intra - capsular Extraction of Cataract”

Starting from Hulen’s device (**Fig. 4**) that needed a 19-l demijohn to create vacuum, smaller and smaller devices were imagined, devices that the surgeon could keep in one hand (**Fig. 5**) [**21**]. This type of instrument was called erysiphake.

Because of the risk for lens luxation when the forceps or erysiphake was used, certain authors have suggested transscleral stabilization first. This technique was described by Dixon in 1853 [**22**] and required the introduction of two needles through the sclera, behind the lens, while the patient was lying in prone position. The lens blocked in the anterior chamber was more easily explanted. Starting from this idea, several complex instruments consisting of one, two or three needles were invented.

**Fig. 6:**
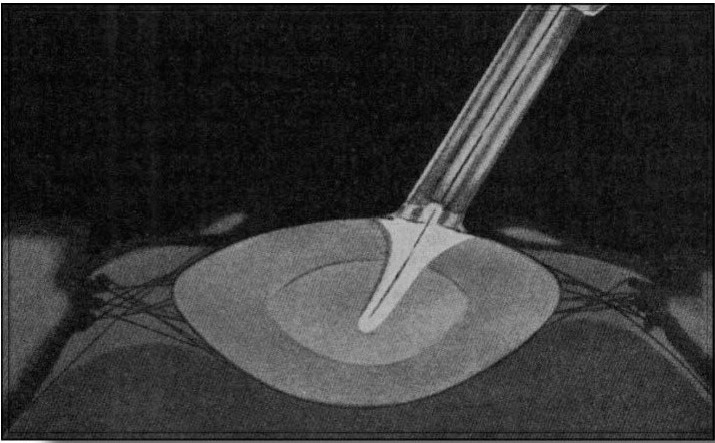
Coagulation of lens using electro-diafaco method. Image reproduced after Khalil M’s. “The extraction of Cataract by the Electro-diafaco Method”

The coagulation effect that diathermy has on the lens proteins, lies at the basis of a new technique of removing subluxated lenses – the electro-diafaco method [**23**]. First described by Lacarrere in 1923, this technique entailed the use of a special device consisting of a manual component (an electrode) and a pedal, which were interconnected through a cable [**22**,**23**]. The manual component was made of glass and contained one or two metal wires. When the pedal was activated, a 90mA electric current passed through them. The metal wires made a 3 mm deep penetration into the lens and had a coagulation effect over 2-3 mm area (**Fig. 6**) [**22**,**23**]. When the manual device is taken out from the eye, the lens strongly adhering to the metal wires is removed.

**Fig. 7:**
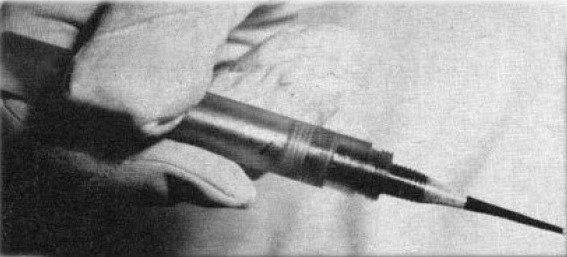
Cryoextractor. Image reproduced after Krwawicz T.’s “Intracapsular Extraction of Intumescent Cataract by Application of Low Temperature”

Opposed to electrodiathermy, another technique employed in severe cases was cryoextraction. The latter was introduced by Krwawicz in 1961 and used low temperatures to remove the lens, diminishing the risk of breaking the anterior capsule [**24**]. The device was named cryoextractor (**Fig. 7**) and looked like a blunt pen made of nickel-plated copper piece mounted on a plastic handle [**24**].

**Fig. 8:**
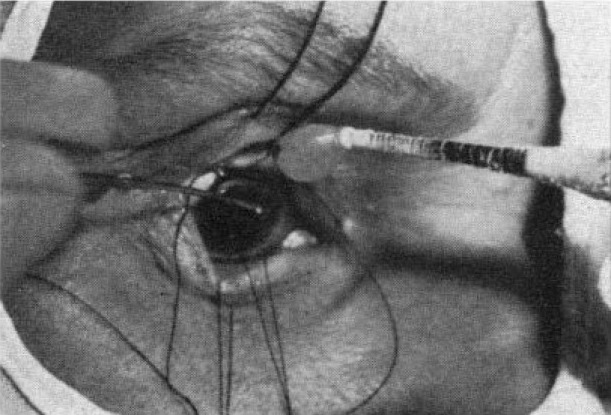
Intraoperatory image of lens removal by cryoextraction. Image reproduced after Krwawicz T.’s “Intracapsular Extraction of Intumescent Cataract by Application of Low Temperature”

The technique implied the following steps: the device was first placed in a silver cylinder containing ethanol, which was placed in a thermal device with carbonic ice and methanol – freezing the cryoextractor to -79 degrees C; the suture threads were put into place and a 180 degrees corneal incision was performed, followed by the incision of the iris and its retraction, between meridians 11 and 1; the upper edge of the lens was gently raised by counter-pressure at the level of inferior cornea and, then, the extractor was applied on the anterior capsule, close to the lens equator, on the 12 o'clock meridian; when the cryoextractor touched the capsule, the lens froze (**Fig. 8**) and was removed by sliding, after breaking the zonular fibers by lateral movements. Starting from this idea, several devices were created, all using a large variety of freezing agents, i.e., liquid nitrogen, phreon, thermoelectric semiconductors.

Since 1998, when Cionni and Osher introduced the modified capsular tension rings [**25**], ectopia lentis surgery has had a significant progress. 

The technique most frequently employed by the authors makes use of such a device. The surgical technique steps are as follows: once the subluxation maximal zone is marked, incisions are made by means of phacoemulsification or simple aspiration, together with capsulorhexis and removal of the lens matter; in the maximal subluxation zone, the conjunctiva is cut at the level of the limbus and then, a sclera pocket is made by means of a crescent knife; at 1.5 mm from the limbus, the straight needle of a 10.0 doubly-enforced prolene suture is introduced into the eye and externalized through the main incision; the needle is passed through the capsular tension ring eyelet, and then the ring is introduced in the lens capsular bag; the straight needle is then passed through the main incision (in a backward insertion movement), and externalized at 1 mm from the insertion penetration site; after the implantation of the lens in the capsular bag, the thread ends are taken out through the scleral pocket and severed; the whole complex bag- artificial lens - ring is centered by pulling the two ends that are then tied up; the operation is concluded by hydrating the incision, the scleral or conjunctival threads being no longer necessary.

## Discussion

Even though historical techniques were considered innovative for their times, there were progressively abandoned due to numerous associated complications. 

These techniques are no longer in use and they should not be reproduced in modern surgery.

“Couching” by luxation of the lens into the vitreous led only to the temporary improvement of visual acuity. The luxation of the opacified lens into the vitreous only increased the light quantity that reached the retina, and not the visual acuity itself. The improvement was temporary because, after a while, complications appeared, i.e., iritis, iridocyclitis, glaucoma or the incomplete luxation of the lens [**10**].

By their conservatory nature, the discission techniques had a lot of supporters who have adapted and modified the initial technique. However, this technique was difficult to use in lenses with zonular weakness and frequent and repeated interventions were required in order to generate the desired effect.

Given the “blind” manipulation and the pressure applied during the intracapsular removal of the lens according to Smith’s technique, it presented many disadvantages, among which the most important were the vitreous loss, the macular edema, and the detachment of the retina [**26**]. According to Kerby’s technique, the mechanical tearing of the zonular fibers minimizes the tractions upon the vitreous base and, consequently, diminishes possible complications.

The techniques requiring the removal of the lens by means of forceps, as well as those using special devices (erysiphake, cryoextractor) that work upon the lens were always difficult to apply in ectopia lentis because of the weakness of the zonular support.

With all the above-mentioned techniques surgery was a success if the removal of the whole lens was attained.

The aim of the current techniques is to keep the internal anatomy of the eye untouched and preserve the capsular bag. These can be achieved using modern instruments and devices that allow the removal of the lens followed by capsular bag strengthening through small incisions.

## Conclusions

Current and old techniques as well, have always aimed at improving the life quality of patients suffering from ectopia lentis. The invention of the rings that tension the capsular bag was, probably, the greatest improvement in the surgery of ectopia lentis. Postoperative prognosis is considerably better, reducing the number of complications and of further surgeries.

**Conflict of Interest statement**

Authors state no conflict of interest.

**Acknowledgements**

None.

**Sources of Funding**

None.

**Disclosures**

None.
